# Obituary

**Published:** 1912-01-06

**Authors:** 


### Obituary.

Dr. Wm. Henry Hubert, of Billingshurst, Sussex, died suddenly in his sixty-seventh year. He was an old St. George's man, and became a member of the Royal College of Surgeons in 1868. He held the appointments of Public Vaccinator and District Medical Officer.

Dr. Leonard Kingsford Cooper has died from septicæmia, contracted while assisting at an operation. Dr. Cooper was only recently qualified, and his sad death at the age of twenty-five is most regrettable.

Dr. William Soper, of Ventnor, Isle of Wight, has died at the age of seventy-six. He was formerly in practice in the South of London, where he held several appointments, including that of Medical Officer to the Jews' Hospital, Norwood. Dr. Soper was vice-president of the Therapeutical Section of the Royal Society of Medicine. He was a student of Guy's Hospital, and qualified in 1861.

Mr. Henry Robert Heather Bigg, of Wimpole Street, has died in his 58th year. Mr. Heather Bigg was a well-known authority on spinal curvature and other deformities, and contributed largely to the literature on these subjects. He was formerly a student of University College Hospital, and qualified at Edinburgh in 1884, later taking the Fellowship of the Royal College of Surgeons there. In 1887 he compiled a report for the Government on the improvements possible in artificial limbs for soldiers. Mr. Heather Bigg was an Associate of the Institute of Civil Engineers.

